# Neutrophil to Lymphocyte Ratio Predicts Adverse Cardiovascular Outcome in Peritoneal Dialysis Patients Younger than 60 Years Old

**DOI:** 10.1155/2020/4634736

**Published:** 2020-05-20

**Authors:** Yingsi Zeng, Zijun Chen, Qinkai Chen, Xiaojiang Zhan, Haibo Long, Fenfen Peng, Fengping Zhang, Xiaoran Feng, Qian Zhou, Lingling Liu, Xuan Peng, Evergreen Tree Nephrology Association, Guanhua Guo, Yujing Zhang, Zebin Wang, Yueqiang Wen, Jiao Li, Jianbo Liang

**Affiliations:** ^1^Department of Nephrology, The Second Affiliated Hospital, Guangzhou Medical University, Guangzhou, China; ^2^Department of Nephrology, Affiliated Dongguan People's Hospital, Southern Medical University, Guangdong, China; ^3^Department of Nephrology, The First Affiliated Hospital of Nanchang University, Nanchang, Jiangxi, China; ^4^Department of Nephrology, Zhujiang Hospital, Southern Medical University, Guangzhou, China; ^5^Department of Nephrology, Jiujiang No. 1 People's Hospital, Jiangxi, China; ^6^Department of Medical Statistics, The First Affiliated Hospital, Sun Yat-sen University, Guangzhou, China; ^7^Department of General Medicine, The Third Affiliated Hospital, Sun Yat-sen University, Guangzhou, China; ^8^Evergreen Tree Nephrology Association, Guangzhou, China; ^9^Department of Cardiology, The Second Affiliated Hospital, Guangzhou Medical University, Guangzhou, China

## Abstract

**Background:**

Neutrophil to lymphocyte ratio (NLR) is a new inflammatory marker; the relationship between NLR and adverse cardiovascular (CV) prognosis has been gradually emphasized in the general population. However, their association in peritoneal dialysis (PD) patients remains unclear.

**Methods:**

From January 1, 2010, to May 31, 2017, a total of 1652 patients were recruited. NLR was categorized in triplicates: NLR ≤ 2.74, 2.74 < NLR ≤ 3.96, and NLR > 3.96. Kaplan-Meier cumulative incidence curve and multivariable COX regression analysis were used to determine the relationship between NLR and the incidence of adverse CV outcome, while a competitive risk model was applied to assess the effects of other outcomes on adverse CV prognosis. Besides, forest plot was investigated to analyze the adverse CV prognosis in different subgroups.

**Results:**

During follow-up, 213 new-onset CV events and 153 CV disease (CVD) deaths were recorded. Multivariable COX regression models showed that the highest tertile of NLR level was associated with increased risk of CV events (HR = 1.39, 95%CI = 1.01‐1.93, *P* = 0.046) and CVD mortality (HR = 1.81, 95%CI = 1.22‐2.69, *P* = 0.003), while compared to the lowest tertile. Competitive risk models showed that the differences in CV event (*P* < 0.001) and CVD mortality (*P* = 0.004) among different NLR groups were still significant while excluding the effects of other outcomes. In subgroups, with each 1 increased in the NLR level, adjusted HR of new-onset CV event was 2.02 (95%CI = 1.26 − 3.23, *P* = 0.003) and CVD mortality was 2.98 (95%CI = 1.58 − 5.62, *P* = 0.001) in the younger group (age < 60 years).

**Conclusions:**

NLR is an independent risk factor for adverse CV prognosis in PD patients younger than 60 years old.

## 1. Introduction

Chronic kidney disease (CKD) is the main disease that threatens public health. To this day, peritoneal dialysis (PD) remains the primary alternative to end-stage renal disease. Cardiovascular (CV) events are the main complications of PD patients [1]. According to reports, CV disease (CVD) death accounts for a substantial part of death in dialysis patients, and the CVD mortality of dialysis patients is 10 to 20 times higher than that of the general population [2]. Therefore, early prevention and diagnosis of CV events are of great significance for PD patients.

There are many reasons for the increased risk of CV events in PD patients, one of which is the increase in inflammation. Neutrophil to lymphocyte ratio (NLR) is regarded as a new inflammatory marker which has been getting widely used in CVD. Neutrophils reflect the state of inflammation, while lymphocytes have been shown to be associated with the stress and nutritional status [3]. NLR can be used as a new indicator of microinflammation in vivo. Microinflammation is a strong predictor of CVD in dialysis patients, which further accelerates the progress of atherosclerosis [4].

NLR has been extensively studied in many CVD. Accumulated evidences have pinpointed that NLR plays a key role in predicting the adverse CV prognosis of patients with acute coronary syndrome, peripheral vascular disease, ischemic encephalopathy, and atrial fibrillation [5–7]. Systematic review indicated that NLR was not only significantly associated with all adverse CV events but also predicted adverse CV prognosis in general population [8]. Besides, NLR has been proved to be a perfect indicator of the increased CV events in patients with CKD stages 3-5 [9]. Recently, the relationship between NLR and adverse CV prognosis has been gradually emphasized in the general population and patients with coronary heart disease. However, little known of its value in the interaction between NLR and adverse CV prognosis in patients undergoing PD. Therefore, we aim to investigate the association between NLR and adverse CV prognosis in PD patients.

## 2. Methods

### 2.1. Subjects

**Figure 1:**
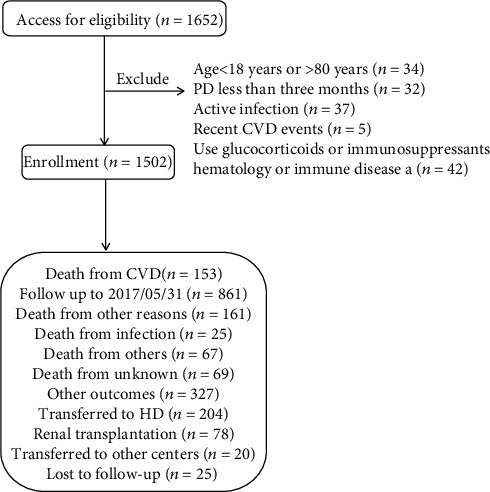
Flow chart including patient enrollment and outcomes. Note: recent CVD events, CVD events occurred between the initiation of PD and the first time follow-up.

From January 1, 2010, to May 31, 2016, a total of 1652 patients were recruited from four PD centers. One hundred and fifty patients were excluded for the following reasons: age younger than 18 years or older than 80 years (*n* = 34), PD was maintained for less than 3 months (*n* = 32), recent CV event (*n* = 5), history of hematological or autoimmune disease and taking glucocorticoids or immunosuppressants (*n* = 43), and clinical evidence of active infection (*n* = 37). Above patients were excluded because those factors may affect NLR level. Finally, 1502 patients were enrolled (Figure 1). During a median follow-up period of 37.1 months, 213 new-onset CV events and 153 CVD deaths were recorded. Recent active infection is defined as hospitalization for pneumonia or peritonitis one month before the return visit. Recent CV disease is defined as adverse CV events from the beginning of PD to the return visit. Written informed consent was not required because we retrospectively collected available medical records in the hospital.

All patients were followed up until death, transferring to hemodialysis therapy, transferring to kidney transplantation, transferring of care from four centers, lost to follow-up, or censoring on May 31, 2017. The primary outcome was CVD mortality, and the secondary outcome was new-onset CV events.

### 2.2. Clinical Data

The baseline demographic data included center, age, gender, complications (hypertension, diabetes mellitus, and CVD history), and medication history (including calcium channel blockers (CCB), diuretics, and insulin). Baseline data were collected within 3 months of the initiation of PD. Clinical biochemical indicators included body mass index, systolic blood pressure, diastolic blood pressure, white blood cell count, neutrophil count, lymphocyte count, hemoglobin, serum albumin, creatinine, urea nitrogen, total cholesterol, total triglyceride, uric acid, fasting glucose, sodium, chlorine, calcium, potassium and phosphorus, total *Kt*/*V*, and residual renal function. CV events are defined as recording any of the following conditions in the patient's medical records after the initiation of PD: coronary heart disease, coronary atherosclerotic heart disease, acute myocardial infarction, cardiac arrest, cerebrovascular accident, stroke, and congestive heart failure. If the patient died because of the above causes, it is considered to be CVD death. Patients who reported current use of insulin or oral hypoglycemic agents and/or who had a clinical diagnosis of type 1 or type 2 diabetes mellitus were considered to have diabetes mellitus. Hypertension was recorded if the patient took antihypertensive drugs or had 2 separate blood pressure measurements ≥ 140/90 mmHg.

Laboratory measurements were obtained using standard methods in the clinical laboratory. Total *Kt*/*V* was calculated using PD Adequest software 2.0 (Baxter, Deerfield, IL). Medicine use was recorded based on prescriptions. The patients returned to these centers for quarterly evaluation, and trained nurses interviewed the patients by telephone monthly to assess general conditions.

### 2.3. Statistical Analysis

Neutrophil to lymphocyte ratio was categorized in triplicates: NLR ≤ 2.74, 2.74 < NLR ≤ 3.96, and NLR > 3.96. By way of normality test, the continuous variable data were all skewed distribution data. The values for skewed variables were described as median (25th, 75th percentile), and categorical data were given as percentages. Differences among the NLR groups were tested using chi-square test for categorical variables, Kruskal–Wallis test for skewed continuous variables. A univariable logistic regression model was used to examine the association between patients' characteristics and new-onset CV event as well as CVD mortality with lower category as reference, and then a multivariable logistic regression was used to examine patients' characteristics with predictive odds of high CV event incidence and CVD mortality, which were adjusted for covariates with (*P* < 0.05 in a univariable logistic analysis). Incidence of new-onset CV event and CVD mortality was calculated using the Kaplan-Meier curve and differences among distributions were assessed by Log rank test. COX regression models were used to evaluate the relationship among NLR groups with the incidence of new-onset CV event as well as CVD mortality in patients undergoing PD, initially without adjustment and subsequently adjusting for several groups of covariates. The multivariable COX regression model was constructed using eligible covariates that demonstrated significant or near-significant association with incidence of new-onset CV event as well as the CVD mortality (*P* < 0.05) on multivariable logistic analysis or characteristics (*P* < 0.05) listed in Table 1 or for importance of clinical concern.

In COX regression models, time at risk was from study entry until new-onset CV event, death, transferring to hemodialysis therapy, transferring to kidney transplantation, transferring of care from four centers, lost to follow-up, or the end of study on May 31, 2017. Moreover, the interaction between subgroup variables of interest including sex, age, and history of diabetes mellitus and NLR groups were examined by performing a formal test of interaction. Forest plot was used to represent the relationship between NLR and the new-onset CV event as well as CVD mortality in each subgroup. Excluding the effects of other outcomes such as death or death except cardiovascular cause, transfer to hemodialysis therapy, transfer to kidney transplantation, transfer of care from four centers, and lost to follow-up, the differences in the incidence of CV event and CVD mortality among different NLR groups were assessed using a competitive risk model. Due to the data of continuous variables in skewed distribution, missing covariate values were filled by median. Statistical analysis is completed by SPSS 23.0 and R software (version R, 3.6.1, www.r-project.org). All tests were performed bilaterally, and *P* < 0.05 was considered to be statistically significant.

## 3. Results

### 3.1. Participants

During a median follow-up period of 37.1 months, 213 (14.2%) new-onset CV events and 153 (10.2%) CVD deaths were recorded. Baseline characteristics of the cohort categorized according to NLR level are summarized in Table 1. As shown in Table 1, the median age is 51 (41, 62), of which 852 are male and 650 are female. 343 (22.8%) patients had a history of diabetes, 985 (65.6%) patients had a history of hypertension, and 232 (15.4%) patients had a history of CVD. Median NLR value was 3.3 (2.5, 4.5) for all patients.

### 3.2. Risk Factors for Higher Incidence of New-Onset CV Event and Higher CVD Mortality in PD Patients

The significant risk factors for patients with the higher incidence of new-onset CV event as well as CVD mortality were given in Table 2 by adjusting for covariates (*P* < 0.05 univariable logistic regression). Higher incidence of new-onset CV event was associated with male, older age, higher blood glucose level, and history of hypertension (Table 2). Higher CVD mortality was associated with older age, history of hypertension, history of diabetes, history of CVD, and center 4 (Table 2).

### 3.3. NLR Associated with the New-Onset CV Event and CVD Mortality in PD Patients

Associations of NLR with new-onset CV event and CVD mortality with defined models (with the lowest tertile as the reference group) are listed in Table 3. Multivariate COX regression showed that elevated NLR was an independent risk factor for CV event as well as CVD mortality in patients undergoing PD after adjusting for complications, age, sex, BMI and biochemical examination. For each unit of NLR increase, the risk of new-onset CV event increased by 1.39 times and the risk of CVD mortality increased by 1.81 times (Table 3).

**Figure 2:**
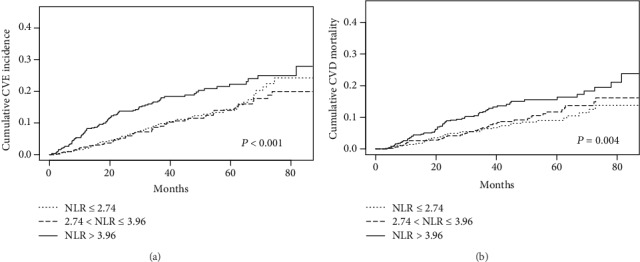
Competitive risk models for CV prognosis and other competing events. (a) Estimated cumulative incidence curves between the new-onset CV event and other competing events for each type of NLR level. (b) Estimated cumulative incidence curves between CVD mortality and other competing events for each type of NLR level. The cumulative incidence curves for different NLR groups are highly significant for the new-onset CV events (*P* < 0.001) and the CVD mortality (*P* = 0.004), but they are not statistically different for the occurrence of other events.

The cumulative incidence curves demonstrated significant differences in different NLR groups for the new-onset CV events (*P* < 0.001), but they are not statistically different for the occurrence of other events (death (coded as 2) (*P* = 0.912), transferring to hemodialysis therapy (coded as 3) (*P* = 0.687), transferring to kidney transplantation (coded as 4) (*P* = 0.481), transferring to other center (coded as 5) (*P* = 0.810), and being lost to follow-up (coded as 6) (*P* = 0.526)) (Figure 2(a)). Meanwhile, the cumulative mortality curves for different NLR groups are strongly significant for the CVD mortality (*P* = 0.004), but they are not statistically different for the occurrence of other events (death except CV cause (coded as 2) (*P* = 0.983), transferring to hemodialysis therapy (coded as 3) (*P* = 0.076), transferring to kidney transplantation (coded as 4) (*P* = 0.459), transferring to other centers (coded as 5) (*P* = 0.810), and being lost to follow-up (coded as 6) (*P* = 0.518)) (Figure 2(b)).

### 3.4. NLR Associated with the New-Onset CV Events and CVD Mortality in Different Subgroups

**Figure 3:**
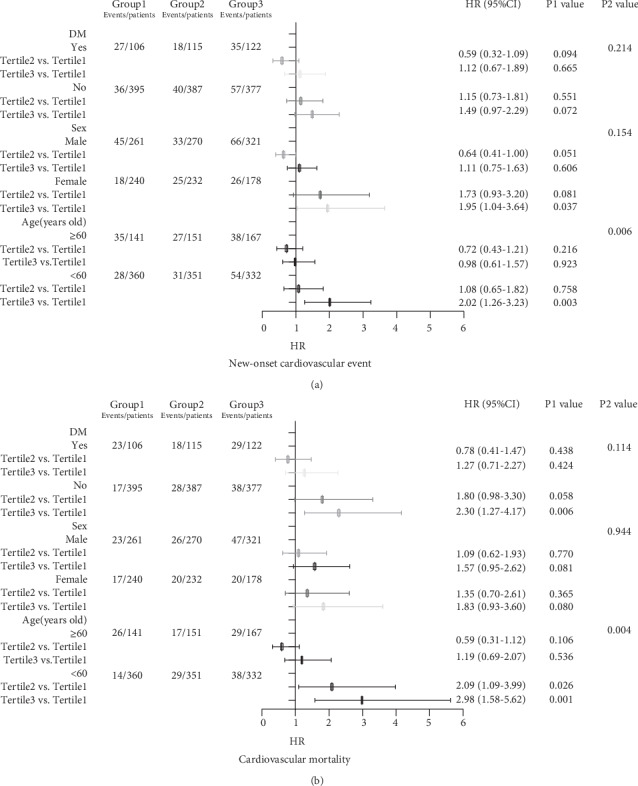
Forest plot of relationship between NLR and CV prognosis in different subgroups. (a) Forest plot of relationship between NLR and the new-onset CV event in different subgroups. (b) Forest plot of relationship between NLR and CVD mortality in different subgroups. Note: The P1 value corresponds to the relationship between NLR and the new-onset CV event or cardiovascular mortality in different subgroups. The P2 value corresponds to the interaction test between the NLR and the subgroups variable of interest.

The forest plot indicated that there was an interaction between age and NLR no matter in the incidence of new-onset CV event or CVD mortality. Nevertheless, no interaction was found in other subgroups (Figure 3). In the younger group (age < 60 years), with each 1 increase in NLR level, adjusted HR of the incidence of new-onset CV event was 2.02 (95%CI = 1.26 − 3.23, *P* = 0.003), and adjusted HR of CVD mortality was 2.98 (95%CI = 1.58 − 5.62, *P* = 0.001). In another group (age ≥ 60 years), the association was not observed (*P* = 0.923, *P* = 0.536) (Table 4).

**Figure 4:**
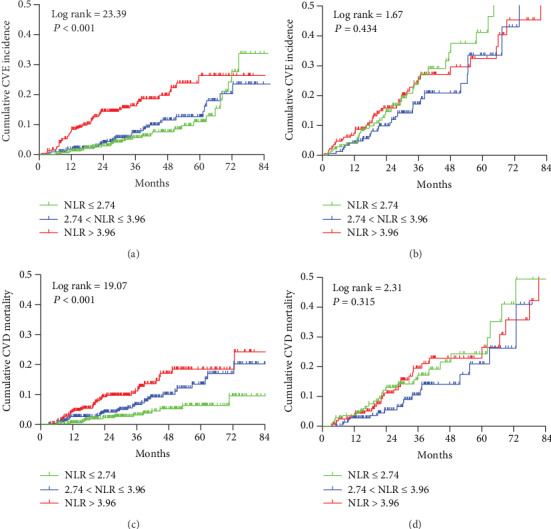
Cumulative incidence curves for CV prognosis by category of NLR in different subgroups. (a) Cumulative incidence curves for the new-onset CV event by category of NLR in the group younger than 60 years old. (b) Cumulative incidence curves for the new-onset CV event by category of NLR in the group older than 60 years old. (c) Cumulative incidence curves for CVD mortality by category of NLR in the group younger than 60 years old. (d) Cumulative incidence curves for CVD mortality by category of NLR in the group older than 60 years old. The curves were constructed using the Kaplan-Meier method and compared using the Log rank test.

The Kaplan-Meier cumulative incidence curve demonstrated that in the group younger than 60 years, the higher NLR group had greater incidence of new-onset CV event (Log rank test *P* < 0.001) (Figure 4(a)) and CVD mortality (Log rank test *P* < 0.001) (Figure 4(c)) than the lower NLR group. But in groups older than 60 years, the Kaplan-Meier curve showed no difference in the incidence of CV event (Log rank test *P* = 0.434) (Figure 4(b)) or CVD mortality (Log rank test *P* = 0.315) (Figure 4(d)).

## 4. Discussion

The main finding of our study is that the NLR is strongly associated with the new-onset CV event and CVD mortality in PD patients younger than 60 years old, after adjusting for complications, sex, BMI, and biochemical examination. In another group (age ≥ 60 years), no association was observed. It may be the first time that NLR has been found to be associated with adverse CV outcomes in dialysis patients of different age.

Prior evidence has shown that elevated NLR was related to higher incidence of adverse CV events in the general population and CVD patients. Angkananard et al. [8] conducted a systematic review, which pointed out that high NLR was associated with all adverse CV events in the general population. Yan et al. [10] have found a significant positive correlation of NLR with major CV events (MCE) in elderly chronic heart failure patients. Paquissi demonstrated the potential utility of NLR in peripheral arterial disease (PAD) [7]. Nevertheless, there was little research on the relationship between NLR and poor CV outcome in PD patients.

Prior to this study, the association between NLR and CVD has been little investigated in PD patients. An et al. [11] included 138 PD patients and reported that NLR was a strong predictor for all-cause and CVD mortality in PD patients. Abe et al. [12] conducted a prospective study, which enrolled 86 incident Japanese dialysis patients and drew a conclusion that higher NLR was associated with increased risk of CVD in dialysis patients. Lu et al. [13] enrolled 86 patients and suggested that high NLR was able to predict all-cause and CVD mortality in PD patients. However, the number of patients included in those above studies was too small, and the above studies are mostly single-center studies. In our study, we included a total of 1502 PD patients in four centers, which made the conclusions more convincing. Our study found out that NLR was strongly associated with poor CV outcomes in PD patients, after adjusting for complications, age, sex, BMI, and biochemical examination, which was in accordance with other studies.

The possible mechanism was that inflammation has been demonstrated to play an important role in the development of CV events [14]. Leukocyte subtypes, including neutrophils and lymphocytes, are the main immune cells involved in atherosclerosis. Neutrophils secrete inflammatory mediators, which aggravate endothelial damage and promote vascular wall sclerosis. In contrast, lymphocytes regulate the development of atherosclerosis, in which regulatory T cells (a subset of lymphocytes) may have inhibitory effects on atherosclerosis [15]. Therefore, the ratio of NLR has been proposed as a biomarker, which is a potential predictor of CVD risk and prognosis. Cai et al. [16] divided the brachial-ankle pulse wave velocity (BaPWV) into high and low groups by mean value, using the brachial-ankle pulse wave velocity (BaPWV) to judge the degree of arteriosclerosis, which found out that NLR was independently associated with arterial stiffness in PD patients. In addition, NLR was considered to be a new inflammatory marker, while inflammatory processes have been identified as central to the development of CVD, and increased levels of inflammatory markers have proved to be a predictor of future CV events [17]. Li et al. [18] indicated that elevated NLR was an independent predictor of elevated pulse pressure (PP), left ventricular mass index (LVMI), and intima-media thickness (IMT) in hemodialysis patients, which emerged as a biomarker of CV risk. On the other hand, evidences suggested that lymphocytopenia was a strong predictor of acute myocardial damage and is associated with the prognosis of CVD [19, 20]. Beyond that, the strong connection between low lymphocyte count with malnutrition has been proved to be meaningful [21–23], while malnutrition was recognized as one of the causes of adverse CV outcomes.

This study also found that there was a positive correlation between elevated NLR and adverse CV events in groups younger than 60 years old. It may be the first time that NLR has been found to be associated with adverse CV outcomes in dialysis patients of different age groups, which may indicate that NLR is more likely to reflect the incidence of adverse CV events and lead to adverse CV prognosis in people younger than 60 years old. One of the reasons may be that aging is associated with reduced immune responses [24]. A research have pointed out that the ability of immune cells to respond to cytokine signals decreased with age, regardless of the stimulus dose; the cytokine signaling response in the elderly was systematically reduced [25]. Thus, slight inflammatory changes are not enough to affect the immune response. Some people believe that mild inflammation is beneficial and is able to upgrade the anti-inflammatory system in vivo without overwhelming them [26]. Therefore, the inflammatory response in the elderly is also weaker than that in the young when stimulated by harmful factors, which is also consistent with our clinical phenomena. After being infected, the immune response and stress state such as fever of young people will be stronger than that of the elderly. This may be another reason for the results of this article. Anyway, the possible mechanisms underlying the relationship between NLR and adverse CV events in different age groups are poorly understood and need to be further explored.

Our data indicated CCB associated with the incidence of new-onset CV events in PD patients, which is still controversial now. In the general population, studies have shown that CCB use was effective for CV event prevention [27, 28]. On the contrary, other articles suggest that short-acting CCB take associates with higher risk of acute CV events and hospitalizations [29]. Besides, the guidelines indicate that CCB may be harmful in cases of heart failure with reduced ejection fraction (usually left ventricular systolic dysfunction) [30]. Moreover, PD population is different from the general population. In this study, CCB associated with the incidence of new CV events might be due to patients suffering from primary hypertension; also, the influence of confounding factors could not be completely excluded in logistic regression analysis. To our knowledge, there are few studies focusing on CCB and poor CV prognosis in PD patients and a multicenter prospective study is needed for further clarification.

Our research has some strengths. Firstly, we have a large sample of studies, including a total of 1502 patients in four centers. Further, the results have been adjusted for complications, sex, age, BMI, and biochemical examination. What is more, we examined the difference in NLR between different age groups in patients with PD. It is the first time that NLR has been found to be associated with adverse CV outcomes in PD patients of different age groups.

There are also some limitations in this study. First of all, this is a retrospective study. The results of this study can only explain the association between NLR and adverse CV prognosis in patients undergoing PD and cannot confirm the causal relationship. Secondly, NLR values are baseline data and there is a lack of follow-up data. In addition, due to the lack of data, we cannot compare with other inflammatory indicators (such as CRP, PCT). Finally, due to lack of relevant data of echocardiography in our database, we failed to perform statistical analysis on the relationship of echocardiographic characteristics and adverse CV outcomes.

## 5. Conclusion

This study suggests that elevated NLR is associated with adverse CV prognosis in PD patients younger than 60 years old. NLR is easy to obtain and inexpensive. It is a new biomarker for predicting CVD risk in patients undergoing PD. The relationship between NLR and adverse CV events in different age groups needs to be confirmed in larger multicenter prospective study.

## Figures and Tables

**Table 1:** Baseline characteristics of the study population.

	Total (*n* = 1502)	Group1 NLR ≤ 2.74 (*n* = 501)	Group2 2.74 < NLR ≤ 3.96 (*n* = 502)	Group3 NLR > 3.96 (*n* = 499)	*P* value
No. of C1/C2/C3/C4	313/794/36/359	123/249/9/120	125/247/7/123	65/298/20/116	<0.001
No. of men/women	852/650	261/240	270/232	321/178	<0.001
NLR	3.3 (2.5, 4.5)	2.2 (1.8, 2.5)	3.3 (3.0, 3.6)	5.4 (4.5, 7.0)	<0.001
Demographics					
Age (y)	51.0 (41.0, 62.0)	49.0 (39.0, 61.0)	50.5 (41.0, 61.0)	53.0 (43.0, 63.0)	0.008
BMI (kg/m^2^)	22.1 (20.1,24.2)	22.1 (20.0, 24.2)	22.1 (20.3, 24.4)	22.1 (20.1, 24.2)	0.400
Comorbid					
Systolic BP (mmHg)	146.0 (132.0, 164.0)	146.0 130.0, 160.0)	146.0 (134.0, 161.0)	149.0 (134.0, 170.0)	<0.001
Diastolic BP (mmHg)	86.0 (78.0, 95.0)	86.0 (78.0, 95.0)	86.0 (79.0, 95.0)	86.0 (78.0, 96.0)	0.537
Hypertension	985 (65.6%)	310 (61.9%)	319 (63.5%)	356 (71.3%)	0.004
Diabetes mellitus	343 (22.8%)	106 (21.2%)	115 (22.9%)	122 (24.4%)	0.463
Cardiovascular disease	232 (15.4%)	68 (13.6%)	77 (15.3%)	87 (17.4%)	0.239
Treatments					
CCB	1104 (73.5%)	369 (73.7%)	367 (73.1%)	368 (73.7%)	0.970
Loop diuretic	100 (6.7%)	32 (6.4%)	30 (6.0%)	38 (7.6%)	0.557
Insulin	231 (15.4%)	75 (15.0%)	71 (14.1%)	85 (17.0%)	0.427
Laboratory variables					
WBC (10^9^/L)	6.1 (4.9, 7.6)	5.3 (4.3, 6.6)	6.1 (5.0, 7.3)	6.9 (5.7, 9.0)	<0.001
Neutrophil (10^9^/L)	4.1 (3.1, 5.2)	3.0 (2.4, 3.8)	4.1 (3.5, 4.8)	5.3 (4.2, 6.9)	<0.001
Lymphocyte (10^9^/L)	1.2 (0.9, 1.6)	1.5 (1.2, 1.9)	1.2 (1.0, 1.5)	0.9 (0.7, 1.2)	<0.001
Hemoglobin (g/L)	87.0 (74.0, 100.0)	89.0 (76.0, 103.0)	87.0 (75.0, 101.0)	83.0 (70.0, 95.0)	<0.001
Albumin (g/L)	34.6 (31.1, 37.9)	34.9 (31.4, 38.4)	34.7 (31.3, 37.4)	34.2 (30.7, 37.6)	0.046
Creatinine (*μ*mol/L)	708.1 (543.8, 931.1)	699.0 (543.5, 898.5)	713.0 (542.0, 932.7)	728.0 (546.0, 948.8)	0.606
Urea nitrogen (mmol/L)	20.5 (15.5, 26.8)	19.5 (15.2, 25.4)	20.2 (15.3, 26.4)	21.8 (16.3, 29.4)	<0.001
Uric acid (mmol/L)	428.0 (356.0, 508.3)	428.0 (356.0, 511.5)	428.0 (355.0, 504.0)	428.0 (360.0, 504.0)	0.891
FBG (mmol/L)	4.7 (4.1, 5.5)	4.6 (4.1, 5.4)	4.7 (4.1, 5.5)	4.8 (4.1, 5.6)	0.033
Cholesterol (mmol/L)	4.2 (3.5, 5.0	4.3 (3.7, 5.0)	4.2 (3.5, 4.9)	4.2 (3.4, 4.9)	0.009
Triglycerides (mmol/L)	1.3 (1.0, 1.8)	1.3 (1.0, 1.8)	1.3 (1.0, 1.8)	1.3 (0.9, 1.7)	0.029
Sodium (mmol/L)	140.1 (138.0, 142.3)	140.6 (138.3, 142.5)	140.0 (138.0, 142.9)	140.0 (137.7, 142.0)	0.017
Chlorine (mmol/L)	103.0 (99.4, 107.0)	103.2 (99.9, 107.0)	103.0 (99.3, 106.8)	102.8 (99.0, 107.0)	0.103
Calcium (mmol/L)	2.0 (1.9, 2.2)	2.1 (1.9, 2.2)	2.0 (1.9, 2.2)	2.0 (1.8, 2.1)	<0.001
Potassium (mmol/L)	4.1 (3.6, 4.7)	4.2 (3.7, 4.8)	4.1 (3.6, 4.7)	4.1 (3.6, 4.7)	0.226
Phosphorus (mmol/L)	1.7 (1.4, 2.0)	1.7 (1.4, 2.0)	1.7 (1.3, 2.0)	1.7 (1.4, 2.1)	0.005
Total *Kt*/*V*	2.2 (1.8, 2.6)	2.2 (1.8, 2.7)	2.2 (1.7, 2.6)	2.2 (1.8, 2.6)	0.099
RRF(mL/min)	3.9 (2.1, 7.2)	3.9 (2.0, 7.6)	3.9 (2.2, 7.1)	3.9 (2.0, 6.8)	0.856

Note: All continuous variables are skewed distribution, the values for continuous variables are given as median (P25, P75). Abbreviations: C1: center 1; C2: center 2; C3: center 3; C4: center 4; NLR: neutrophil lymphocyte ratio; BMI: body mass index; BP: blood pressure; CCB: calcium channel blocker; WBC: white blood cell; FBG: fasting blood glucose; *Kt*/*V*: *K*, dialyzer clearance of urea; *t*, dialysis time; *V*, volume of distribution of urea; RRF: renal residual function.

**Table 2:** Significant risk factors for new-onset CV event and CVD mortality.

Risk factors	Univariable logistic regression		Multivariable logistic regression	
	HR (95% CI)	*P* value	HR (95% CI)	*P* value
New-onset CV event				
Sex (female vs. male)	0.58 (0.43-0.79)	0.001	0.56 (0.40-0.77)	<0.001
Hypertension (yes vs. no)	2.63 (1.83-3.79)	<0.001	1.72 (1.16-2.56)	0.008
Age (per 1 year greater)	1.04 (1.03-1.05)	<0.001	1.04 (1.03-1.05)	<0.001
FBG (mmol/L)	1.13 (1.07-1.20)	<0.001	1.09 (1.03-1.16)	0.005
CCB (yes vs. no)	1.61 (1.12-2.31)	0.010	1.58 (1.04-2.42)	0.034
Diastolic BP (per 1 mmHg greater)	0.99 (0.98-1.00)	0.006		
Creatinine (*μ*mol/L)	0.999 (0.998-1.001)	<0.001		
Phosphorus (mmol/L)	0.70 (0.53-0.92)	0.012		
RRF (mL/min)	0.99 (0.98-1.00)	0.032		
Center (C4 vs. C1)	0.50 (0.31-0.81)	0.005		
Diabetes (yes vs. no)	2.35 (1.72-3.20)	<0.001		
CVD disease (yes vs. no)	2.12 (1.50-3.00)	<0.001		
Diuretic (yes vs. no)	1.79 (1.09-2.94)	0.022		
Insulin (yes vs. no)	2.27 (1.61-3.20)	<0.001		
CVD mortality				
Center (C4 vs. C1)	0.40 (0.21-0.75)	0.004	0.49 (0.25-0.94)	0.032
Hypertension (yes vs. no)	3.10 (1.98-4.85)	<0.001	1.72 (1.06-2.79)	0.027
Diabetes (yes vs. no)	3.32 (2.36-4.69)	<0.001	2.10 (1.42-3.10)	<0.001
CVD disease (yes vs. no)	2.59 (1.77-3.79)	<0.001	1.66 (1.07-2.56)	0.024
Age (per 1 year greater)	1.04 (1.03-1.06)	<0.001	1.03 (1.02-1.05)	<0.001
Diastolic BP (per 1 mmHg greater)	0.99 (0.98-1.00)	0.020		
Albumin (g/L)	0.97 (0.94-1.00)	0.039		
Creatinine (*μ*mol/L)	0.999 (0.998-0.999)	<0.001		
Urea nitrogen (mmol/L)	0.97 (0.95-0.99)	0.001		
FBG (mmol/L)	1.08 (1.02-1.15)	0.010		
Chlorine (mmol/L)	1.04 (1.01-1.07)	0.014		
Phosphorus (mmol/L)	0.61 (0.43-0.85)	0.003		
RRF (mL/min)	0.98 (0.96-1.00)	0.014		
Diuretic (yes vs. no)	1.91 (1.10-3.31)	0.022		
Insulin (yes vs. no)	2.41 (1.64-3.55)	<0.001		

Abbreviations: CVD: Cardiovascular disease; C1: center 1; C4: center 4; BP: blood pressure; CCB: calcium channel blocker; RRF: residual renal function; FBG: fasting blood glucose; HR: hazard ratio; CI: confidence interval.

**Table 3:** Relationship between NLR and new-onset CV event as well as CVD mortality.

	Tertile 2		Tertile 3	
	HR (95% CI)	*P*	HR (95% CI)	*P*
New-onset CV event				
Unadjusted	0.93 (0.65-1.33)	0.701	1.72 (1.25-2.37)	0.001
Model 1	0.88 (0.62-1.26)	0.499	1.41 (1.02-1.95)	0.037
Model 2	0.88 (0.62-1.26)	0.483	1.39 (1.01-1.93)	0.046
Model 3	0.88 (0.62-1.26)	0.483	1.39 (1.01-1.93)	0.046
CVD mortality				
Unadjusted	1.18 (0.77-1.81)	0.436	1.98 (1.34-2.93)	0.001
Model 1	1.17 (0.77-1.79)	0.465	1.73 (1.17-2.56)	0.007
Model 2	1.16 (0.76-1.78)	0.495	1.74 (1.17-2.57)	0.006
Model 3	1.19 (0.78-1.82)	0.416	1.81 (1.22-2.69)	0.003

Note: reference group is the low NLR group. Model 1: sex, age, and BMI. Model 2: new-onset CV event: Model 1 plus medical history (diabetes mellitus, hypertension). CVD mortality: Model 1 plus medical history (diabetes mellitus, hypertension, and CVD). Model 3: Model 2 plus albumin, creatinine, urea nitrogen, triglycerides, cholesterol, chlorine, phosphorus, *Kt*/*V*, and RRF. Abbreviations: NLR: neutrophil lymphocyte ratio; HR: hazard ratio; CI: confidence interval; CVD: cardiovascular disease; BMI: body mass index; *Kt*/*V*: *K*, dialyzer clearance of urea; *t*, dialysis time; *V*, volume of distribution of urea; RRF: residual renal function.

**Table 4:** The incidence of new-onset CV event and CVD mortality for per-1 NLR level increase by age.

	New-onset CV event		CVD mortality	
	HR (95% CI)	*P*	HR (95% CI)	*P*
**<**60 years old				
Tertile2	1.08 (0.65-1.82)	0.758	2.09 (1.09-3.99)	0.026
Tertile3	2.02 (1.26-3.23)	0.003	2.98 (1.58-5.62)	0.001
≥60 years old				
Tertile2	0.72 (0.43-1.21)	0.216	0.59 (0.31-1.12)	0.106
Tertile3	0.98 (0.61-1.57)	0.923	1.19 (0.69-2.07)	0.536

Adjusted model: new-onset CV event: sex, BMI, albumin, creatinine, urea nitrogen, triglycerides, cholesterol, chlorine, phosphorus, *Kt*/*V*, RRF, and medical history (diabetes mellitus, hypertension); CVD mortality: sex, BMI, albumin, creatinine, urea nitrogen, triglycerides, cholesterol, chlorine, phosphorus, *Kt*/*V*, RRF, and medical history (diabetes mellitus, hypertension, and CVD).

## Data Availability

The data was obtained with the consent of all centers, and the data in this study are true and reliable.

## Abbreviations

PD: Peritoneal dialysis
CVD: Cardiovascular disease
HD: Hemodialysis
CVE: Cardiovascular event
NLR: Neutrophil lymphocyte ratio
DM: Diabetes mellitus
HR: Hazard ratio
CI: Confidence interval.
